# CC chemokine CCL1 receptor CCR8 mediates conversion of mesenchymal stem cells to embryoid bodies expressing FOXP3+CCR8+ regulatory T cells

**DOI:** 10.1371/journal.pone.0218944

**Published:** 2019-07-17

**Authors:** Nasreen S. Haque, Akaash Tuteja, Niloufar Haque

**Affiliations:** 1 Department of Pathology, New York Medical College, Valhalla, NY, United States of America; 2 Department of Biological Sciences, New York City College of Technology, City University of New York, Brooklyn, NY, United States of America; National Cancer Institute, UNITED STATES

## Abstract

Embryoid bodies (EBs) are three dimensional aggregates of pluripotent stem cells primarily used to investigate morphogenesis and cell toxicity, are also attractive tools in regenerative medicine. While embryonic stem cells (ESCs) and induced pluripotent cells (IPSCs) have been shown to form EBs in mouse, primate and humans, EB formation have not been previously demonstrated in mesenchymal stem cells (MSCs). Here we show that rat MSCs form EBs; which express regulatory T cell (Treg) marker Foxp3 and CC chemokine CCL1 receptor CCR8. We show a novel method for formation of EBs from MSCs under stress and demonstrate that the induction of FoxP3+ CCR8+ EBs is dependent upon CCL1 gradients which mediate cell proliferation, migration and invasion of mTregs. The identification of EBs and novel FoxP3+ CCR8+ regulatory T cells (mTregs) for selective conversion and isolation of bone marrow derived MSCs offers novel avenues for research, diagnosis and treatment.

## Introduction

Inflammatory disorders are on the rise which is a major cause of concern worldwide. Drug based immunosuppressive regimens are effective in various disorders. However, their long term use causes malignancies, toxicity and infections. Stem cell therapy is an option which is rigorously being undertaken by various laboratories and clinical practices. Three types of stem cells which include embryonic stem cells (ESCs), induced pluripotent stem cells (iPSCs) and mesenchymal stem cells (MSCs) are currently being tested in stem cell based therapies. Apart from ethical issues in using ESCs, the propensity for malignancy is the biggest drawback in using iPSCs and ESCs. MSC or regulatory T cell (Treg) based therapy offer alternative immunosuppressive treatments with lesser side effects. In 1970, Friedenstein *et al*. [1] showed that bone marrow contains hematopoietic, non-adherent cells along with a rare population of plastic-adherent cells which had the capacity to form colony forming units or CFUs [2]. These adherent cells were termed mesenchymal stem cells or MSCs. MSCs maintain a quiescent state due to their self-renewal capacity or undergo differentiation. The self-renewal and multi-lineage differential potential of MSCs is an essential component in regeneration after tissue injury. This has made MSCs the ideal choice in cell therapy.

The success in MSCs transplantation is related to their immunosuppressive properties. The mechanism by which MSCs trigger their immune—suppression is by inhibiting CD4+ CD8+ T cells and preferential Treg differentiation [3]. Tregs are fundamental in controlling various immune responses and are characterized by their expression of CD25 (IL2 receptor alpha chain) and FoxP3 and are known to be involved in tolerance to self-antigens. In particular, Tregs are crucial for maintaining tolerance by down regulating undesired immune responses to self and non-self-antigens [4– 5]. It has been also been demonstrated that in the absence of MSCs, Tregs lose their suppressive capacity [6]. CD25+ Foxp3+ mRNA is only detected when MSC and Tregs are in close association which suggests cell–cell contact as a determining factor in Treg expression of FoxP3 [6]. In all, the mechanisms employed by MSCs to inhibit effector T cell proliferation overlap with the mechanisms involved in Treg induction without interfering with Treg function [7]. Tregs inhibit the proliferation of CD4+ T cells, CD8+ T cells and dendritic cells (DCs). Thus, Tregs show similar immune-modulatory effect as those observed for MSCs. However, immunoregulatory functions of MSC are not fixed but rather the result of microenvironment they encounter *in vivo*. Further studies are needed to establish how and wherein these cells have to be administered and how they may function to safely modulate host immune response in vivo in clinical transplant setting.

As MSCs are known to support wound healing and repair by immune suppression, we postulated that MSCs may divert a subpopulation of MSCs towards regulatory T cell (Treg) fate. Here we show that mouse and rat mesenchymal cells form spontaneous FoxP3+ CCR8+ EBs under stress which has immunosuppressive properties. In addition, the aggregation and induction of FoxP3+ CCR8+ EBs is dependent upon the CC chemokine CCL1 and its receptor CCR8CCL1 /CCR8 interactions. We have previously shown that CCL1/ CCR8 may play a role in vascular pathobiology [8]. Our new finding that the chemokine CCl1 and its receptor CCR8 have a functional role in MSC migration and proliferation and is co-expressed with FoxP3+ mTregs indicates its important role in vascular function and wound remodeling. This novel method for selective conversion and isolation of bone marrow derived mesenchymal stem cells (MSCs) to niche specific embryoid bodies (EBs) and generation of MSC derived mTregs offers novel avenues for research, diagnosis and treatment.

## Methods

### I. Cell culture

#### Isolation of mesenchymal stem cells from rat and mouse bone marrow

MSCs were isolated from rat bone marrow as previously described [9] with some modifications. No animal ethics committee was required for this study as all samples were obtained from deceased animals used for training purposes and provided by Comparative Anatomy at the New York Medical College, Valhalla, NY." Ten week old male Sprague-Dawley rats were sacrificed by cervical dislocation and their tibiae and the femur were separated after cutting off the ankle bone. Following sterilization techniques skin, muscle and connective tissues were stripped off to reveal tibia and the femur by scraping the diaphysis of the bone clean then pulling the tissue toward the ends of the bone. The bones were left in 10% ethyl alcohol for a few seconds before proceeding to the next step. After cutting off the ends of the tibia and femur by sharp scissors, a 27-gauge needle was inserted into the bone marrow and flushed with 1X PBS supplemented with 0.5% Penicillin /streptomycin and fungizone (P/S/F). The extracted pulp was collected in a 15-ml tube and spun at 1500 rpm for 15 minutes. After removal of the supernatant the pellet was re-suspended in Minimum Essential Medium (EMEM) (Life Technologies Grand Island, NY) with 10% horse serum and 0.5% Penicillin /streptomycin in 25 cm^2^ tissue culture flask and incubated at 37°C with 5% CO_2_.

For the isolation of mice, FVB/N strain (2months young) was used. These mice are characterized by vigorous reproductive performance and consistently large litters mice The same procedure was followed as described for rat MSCs, however, the final pellet was suspended in Dulbecco Minimum medium (DMEM) (Life Technologies Grand Island, NY) containing 10% horse serum and 0.5% Penicillin /streptomycin/fungizone (P/S/F) in 25 cm^2^ tissue culture flask and incubated at 37°C with 5% CO_2_.

#### Culture of primary cell lines

Human umbilical vein endothelial cells (ECs) and human aortic smooth muscle cells (SMCs) were obtained from American Type Culture Collection (Manassas, VA) and grown according to the manufacturer's instructions. While ECs were cultured in Dulbecco modified Eagle medium (DMEM) as described [10], SMCs were cultured using the Vascular Smooth Muscle Cell Growth Kit [ATCC PCS-100-042][11–12].

#### Isolation of T cells and mTregs

Using aseptic conditions spleen was removed from young (8 week) and old (17 week) FVB/N mice and placed in a plastic dish with sterile PBS. Next the spleen was teased apart with forceps and passed through a stainless steel mesh to obtain a single cell suspension. This suspension was washed with sterile PBS. Red blood cells were removed using mouse Erythrocyte Lysing Kit (R& D Systems Inc.; Minneapolis, MN) following package instructions. The cell pellet was disrupted briefly by “racking” the tube and re-suspending the cells in erythrocyte lysis solution. The cell suspension was vortexed, incubated for 5–10 minutes at room temperature, and the cells were washed with Wash Buffer obtained from the lysing kit. Splenocytes were next suspended in buffer or medium, further enriched for CD4^+^ T lymphocytes cells were counted before further analysis and/or cell culture.

Embryoid bodies (EBs)/mTregs were formed spontaneously in confluent rat derived MSCs when serum was removed. EBs/mTregs also formed when rMSCs were treated with injured SMC/ECs. The cells detached and started aggregating within 24–48 of deprivation. They range from 200 μm to 600 in size. These cells were further characterized for the presence of Treg markers (FoxP3, CD25 and CCR8). Once confirmed for Treg characteristics the Ebs/mTregs were used for subsequent studies.

#### Proteins and antibodies

Recombinant (human CCL1/ I-309; mouse TCA3), goat polyclonal antibody against CCL1, as well as IgG controls were obtained from R&D systems (Minneapolis, MN). Poxvirus MC148 was obtained from Abcam (Cambridge, MA). Anti-CCR8 was generated as described (10). Anti-CCR8 was conjugated using the APC Conjugation Kit (ab201807) which purchased from Abcam. Antibodies against MSC markers PDGFRα (clone αR1) and Purified Mouse Anti-Rat Nestin Clone Rat 401 (RUO) was purchased from BD Biosciences. One Step Staining Human Treg Flow Kit (FOXP3 Alexa Fluor 488/CD25 PE/CD4 PerCP) was purchased from Biolegend. Secondary antibodies Alexa Fluor 633 goat anti–rabbit IgG, Alexa Fluor 633 goat anti–mouse IgG, and Alexa Fluor 633 goat anti–rat IgG were obtained from Molecular Probes.

#### Immunofluorescence

mMSCs/rMSCs were grown into four well TC slides (BD Falcon) until they reached more than 70% confluency. Serum deprived MSCs developed EBs/mTregs under overnight serum deprivation. When cells reached 90–100% confluency spontaneous EBs were formed. To study the modulation by CCL1/CCR8 MSCs were treated with either recombinant CCL1, antibody against CCL1 (>CCL1) overnight at 37° C with 5% CO2. For Treg specific staining of EBs/mTregs conjugated Alexa Fluor 488/CD25(red) PE/CD4 (yellow) PerCP FoxP3 (green), and CCR8 (APC red) were used. The EBs/mTregs were treated with conjugated antibodies for 30 minutes, washed 3 times and grown on culture slides for 24–48 hours and observed under Nikon T 90 microscope. Images were taken at 10x, 20x and 40x magnification.OpenUrl.

#### Time-lapse imaging of migration and invasion in MSC/mTreg SMCs or ECs co-cultures

Human SMCs and ECs were plated separately in a 96-well format and grown overnight. Upon reaching confluency cells were subjected to automated scratch assay using Incucyte plate scratcher. The plates were subsequently was imaged using an Zoom (Essen Bioscience, Ann Arbor, MI) every 2 h for 30 hours. Cells were imaged under phase contrast as well as fluorescence. Analysis was performed using Incucyte’s Imaging software. The IncuCyte ZOOM Scratch Wound assay utilizes the WoundMaker-IncuCyte ZOOM-ImageLock Plate system to analyze both 2D-migration and 3D-invasion in label-free, live cells (Essen Bioscience). Wound Confluence is a report of the confluence of cells within the wound region, given as the percentage of the wound region area occupied by cells. Relative Wound Density(RWD) relies on measuring the spatial cell density in the wound area relative to the spatial cell density outside of the wound area at every time point. The metric is self-normalizing for changes in cell density which may occur outside the wound due to cell proliferation and/or pharmacological effects. Importantly, the RWD metric is robust across multiple cell types as it does not rely on finding cell boundaries.

#### Statistical analysis

Results of data are reported as the mean ± standard deviation. Levels of significance were determined by 2-tailed Student *t* test.

## Results

### Mesenchymal cells (rMSCs) derived from rat bone marrow cells are multipotent

**Fig 1:**
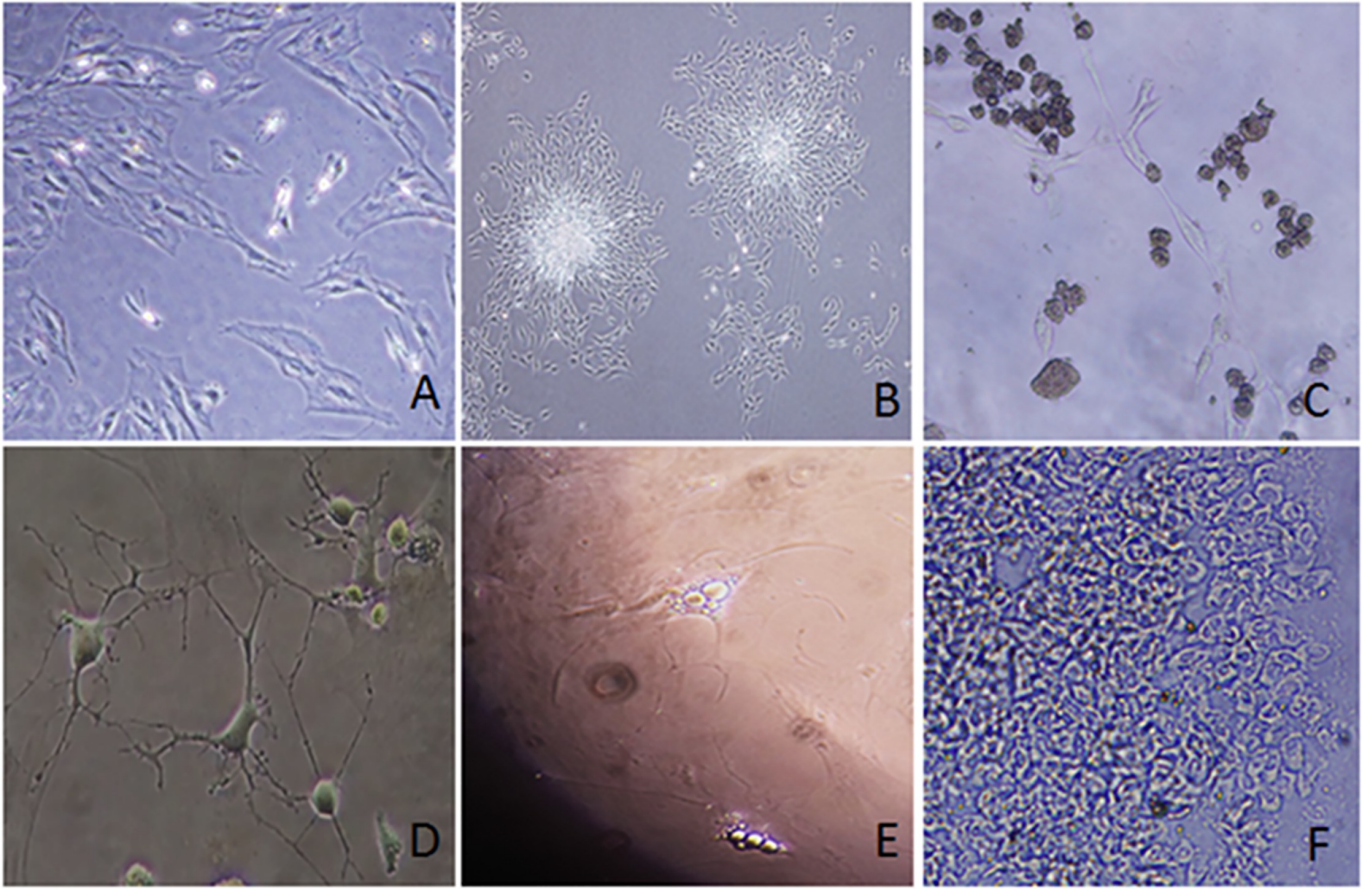
Multipotency of rat mesenchymal stem cells (rMSCs). Undifferentiated rMSCs (1A), forming colonies (1B) or differentiated to mTregs (1C), neurons (1D), adipocytes (1E) and chondrocytes (1F) are shown.

Bone marrow was collected from normal Sprague Dawley rats and mesenchymal cells (rMSCs) were isolated and expanded. These cells demonstrated multipotency. They may remain adherent (Fig 1A) and/or aggregate and form colonies (Fig 1B) with the capacity for differentiation into non-adherent mTregs (Fig 1C), neurons (Fig 1D), adipocytes (Fig 1E) and chondrocytes (Fig 1F). The mTregs obtained here were used for later studies.

### rMSCs form embryoid bodies which express Foxp3 and the chemokine receptor CCR8

**Fig 2:**
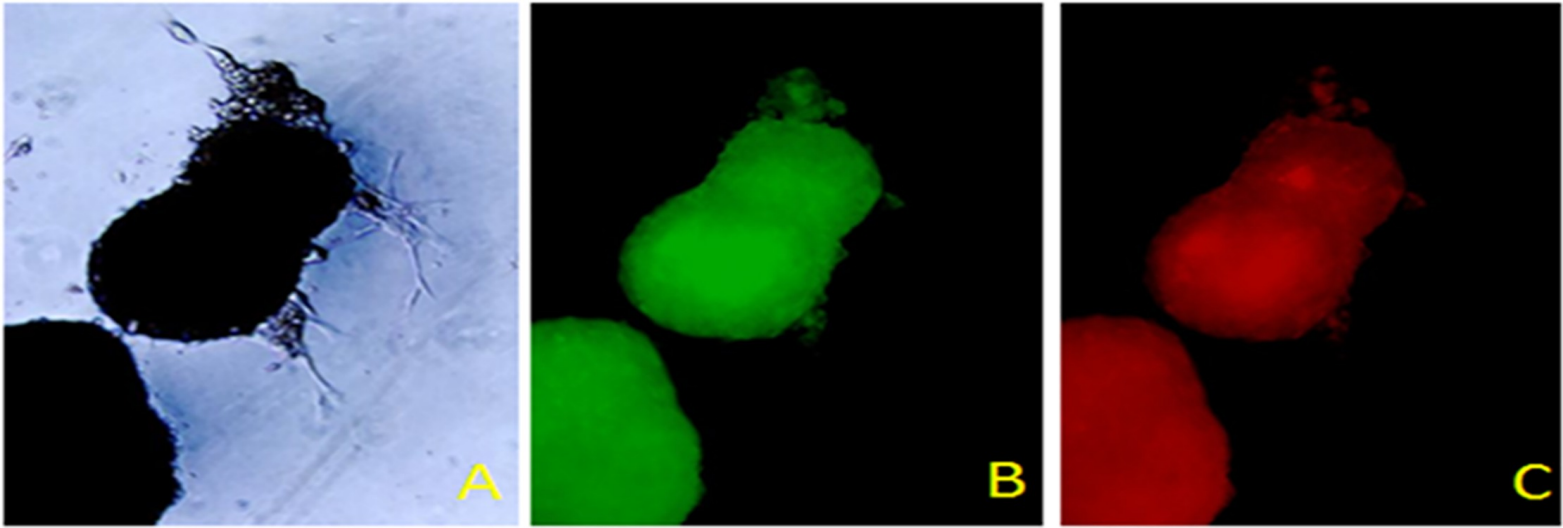
Serum deprived mesenchymal stem cells form embryoid bodies. Embryoid bodies (2A-C) express FoxP3 (green,2B; 10X mag) and CCR8 (red,2C; 10X mag) following serum removal.

We have observed that non adherent rMSCs have a capacity to form spheres or embryoid body (EB) like structures as those reported for embryonic stem cells in the absence of serum (Fig 2). This has not been previously described for MSCs. Following serum removal stressed rMSCs (Fig 2A) formed EBs/mTregs which ranged between 200 μm—600 μm in diameter within 4 weeks. These cells are Foxp3+ (Fig 2B) and express the chemokine receptor CCR8 (Fig 2C). These cells are also CD4+ and CD25+ suggesting that EBs have a Treg like phenotype. The sprouting cells attached to the plastic do not express either FOxP3 or CCR8 suggesting that EBs/mTregs have unique phenotype that has a Treg expression profile.

### mTregs inhibit endothelial cell proliferation and invasion after injury

**Fig 3:**
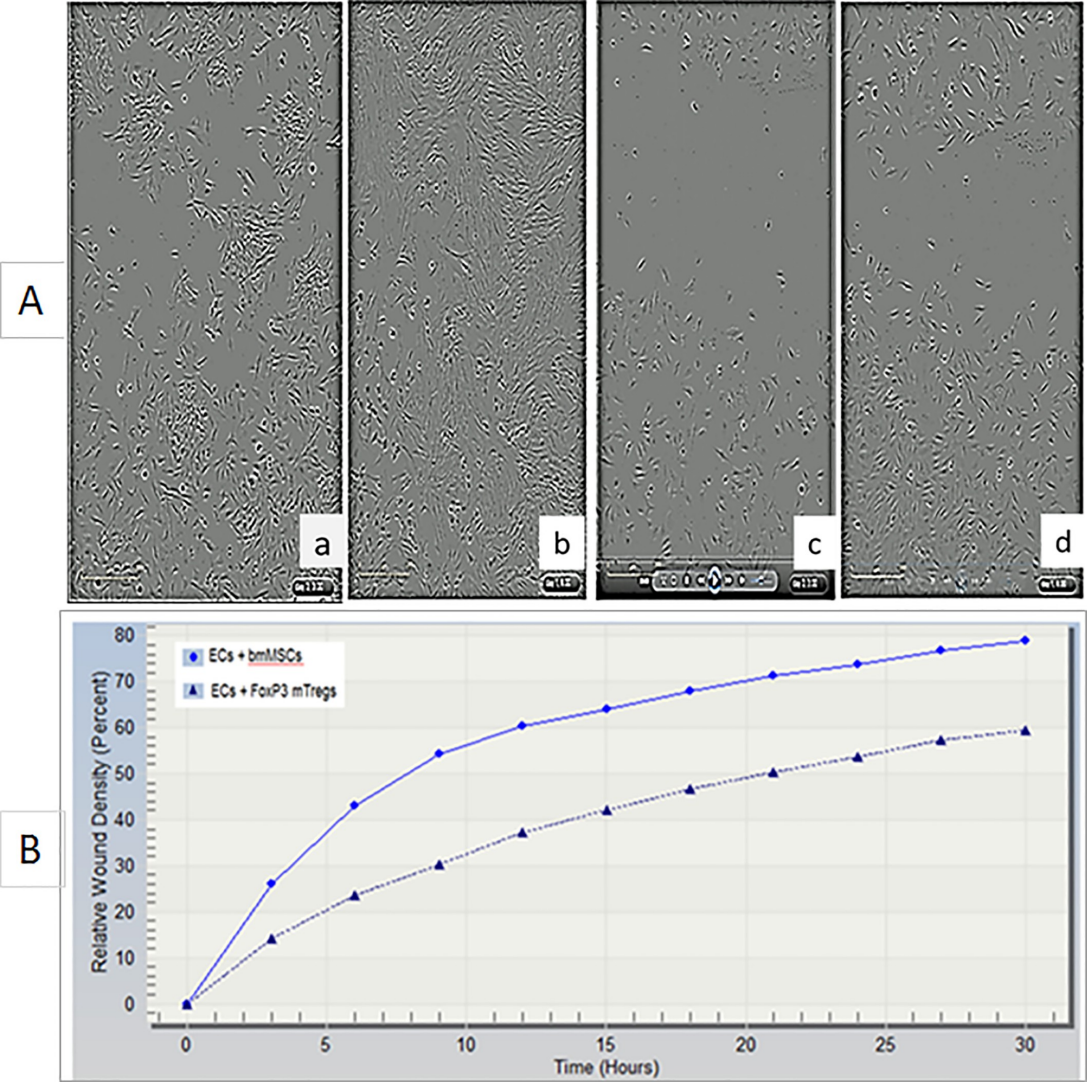
FoxP3+ mTregs inhibits cell migration, invasion and relative wound density (RWD) in injured human endothelial cells (ECs). ECs were treated with MSCs [3A (a, start 3hrs; b, end 30hrs)] or FoxP3+ mTregs [3A (c, start 3hrs; d, end 30hrs)]. FoxP3+ mTregs inhibit RWD in injured human ECs (3B) and S1 Data.

Endothelial cells (ECs) were grown overnight on 96 well plates. They were then subjected to automated scratch assay and immediately co- cultured with rMSCs (Fig 3A–a and b) or mTregs (Fig 3A–c and d) and real time analysis was performed where measurements were taken every 3 hours (S1 Data). Data shown here is at 3 hours (Fig 3A—a and c) and 30 hours (Fig 3A—b and d) after injury. While in rMSC treated ECs the wound was completely covered by 30 hours but not in cells treated with mTregs. This demonstrated that mTregs had an anti- proliferative capacity which may lead to an immunosuppressive function in ECs. This was further confirmed by the analysis of Relative wound confluency (RWC) and relative wound density (RWD) (Fig 3B) in these cells. The RWD is observed to decrease overtime in the presence of mTregs compared to MSC alone (Fig 3B) and S1 Data.

### mTregs inhibit smooth muscle cell proliferation and invasion after injury

**Fig 4:**
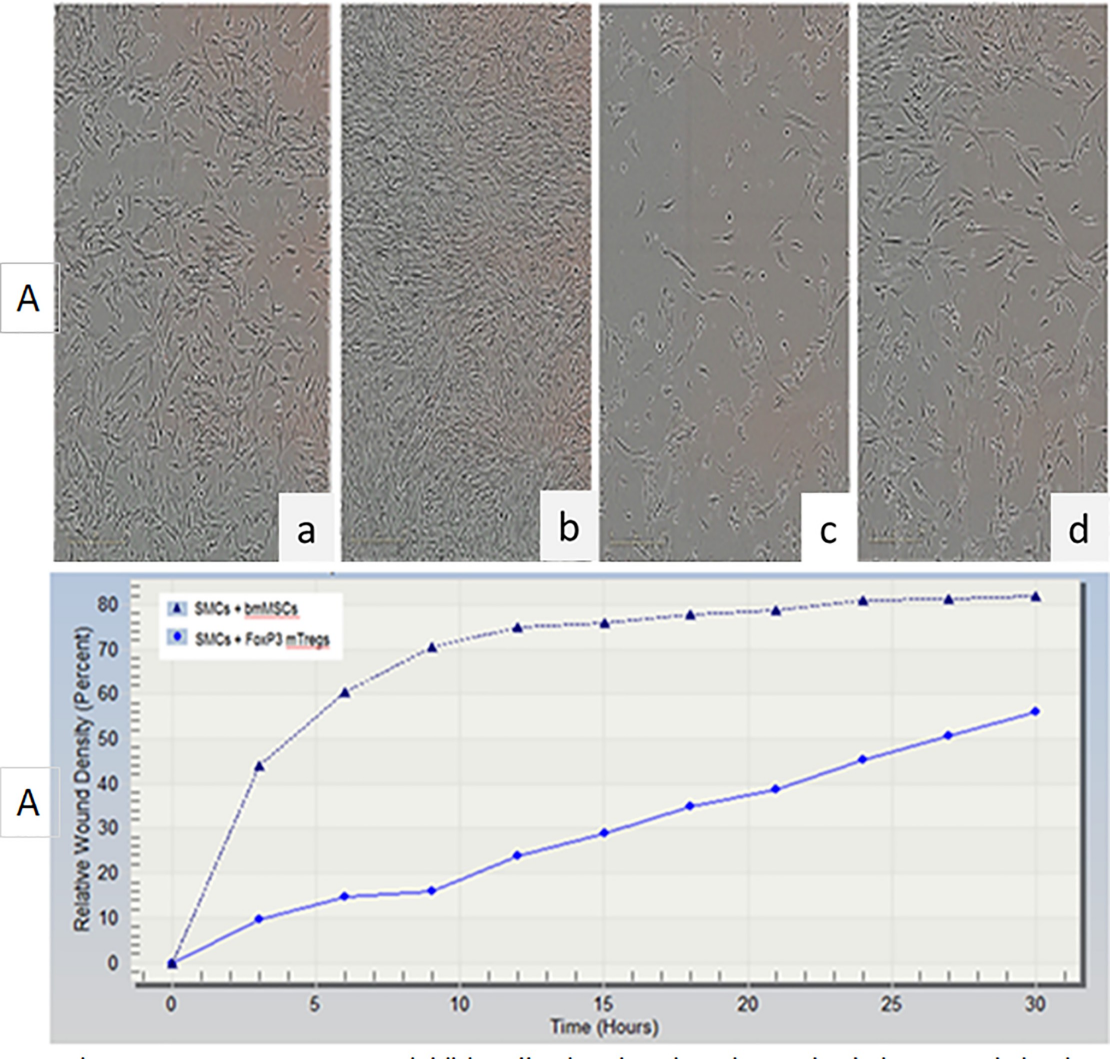
FoxP3+ mTregs inhibit cell migration, invasion and relative wound density (RWD) in injured human smooth muscle cells (hSMCs). hSMCs were treated with MSCs [4A (a, start 3hrs; b, end 30hrs)] or FoxP3+ mTregs [4A (c, start 3hrs; d, end 30hrs)]. FoxP3+ mTregs inhibit RWD in injured hSMCs (4B) and S2 Data.

Vascular smooth muscle cells (VSMCs) were grown on 6 well plates overnight. They were then subjected to automated scratch assay and immediately co- cultured with rMSCs (Fig 4 –a and b) or mTregs (Fig 4 –c and d) and subjected to real time analysis and measurements were taken every 3 hours (S2 Data). Data shown here is at 3 hours (Fig 4 –a and c) and 30 hours (Fig 4 –b and d) after injury. In rMSC treated cells the wound was completely covered by 30 hours but not in cells treated with mTregs. This demonstrated that mTregs had an anti-proliferative which may lead to an immunosuppressive function in SMCs. This was further confirmed by the analysis of Relative wound confluency (RWC) and relative wound density (RWD) (Fig 4B) in smooth muscle cells. The RWD is observed to decrease overtime in the presence of mTregs compared to MSC alone (Fig 4B) and S2 Data

### Mouse mesenchymal stem cells (mMSCs) derived embryoid bodies formation is dependent upon CCL1/CCR8 interaction

**Fig 5:**
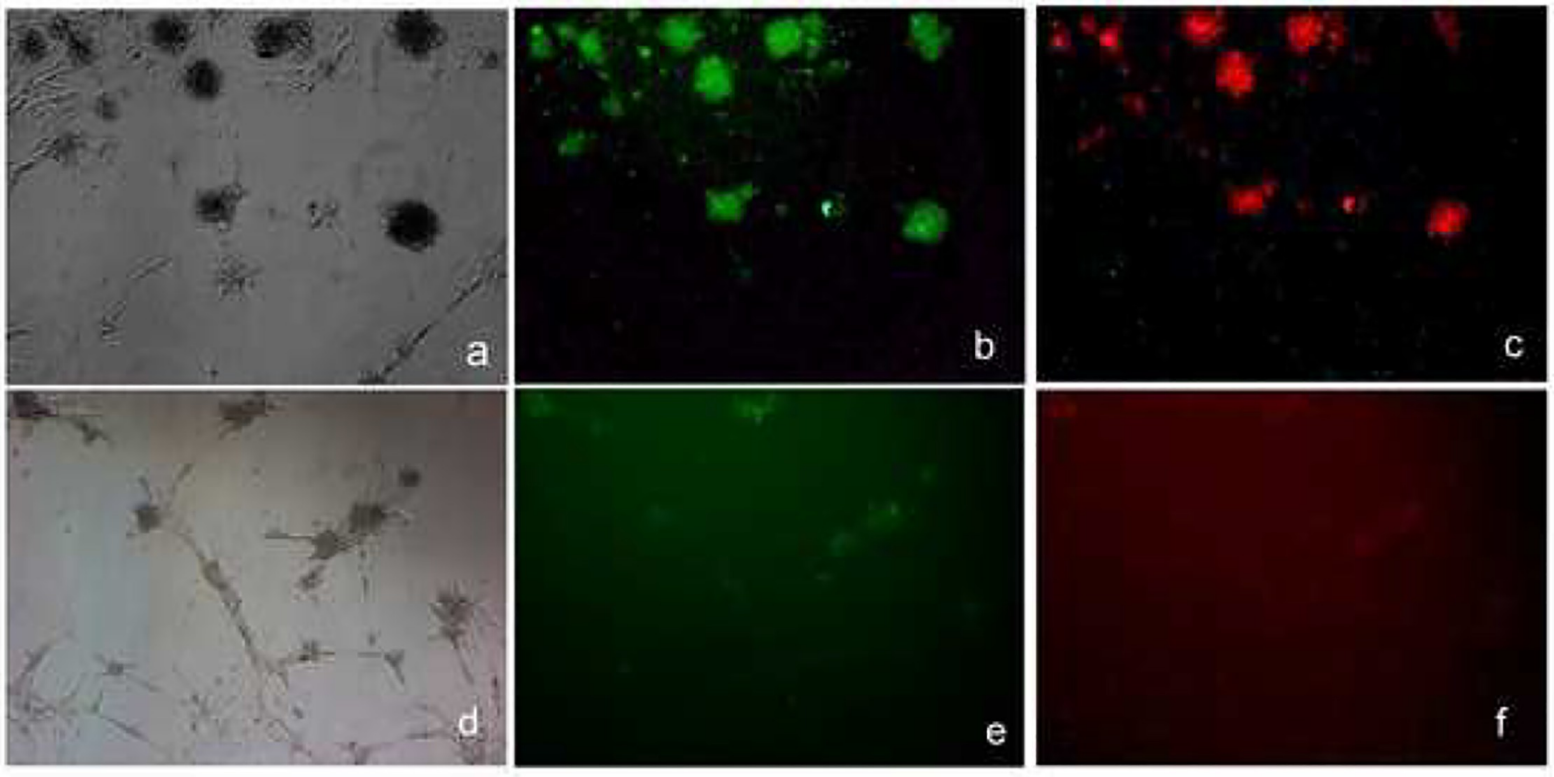
Anti CCL1 inhibits the expression of CCR8 and FoxP3 in mouse mesenchymal stem cells (mMSCs). Untreated (5a,-c) and anti-CCL1 treated (5d,-,f) cells were subjected to immunoflourescence with antibodies against FoxP3 (green;5b and e) or CCR8 (red;5c and f).

In order to see the role of CCL1/CCR8 in the EB formation mMSCs were either treated overnight with anti CCL1 (>CCL1) [Fig 5D–5F] or left untreated (Fig 5A–5C) and subjected to immunofluorescence with antibodies against FoxP3 (Fig 5B and 5E; green) or CCR8 (Fig 5C and 5F; red). It was seen that >CCL1 inhibited the expression of CCR8 and to a lesser extent FoxP3 expression suggesting a role of the CCL-1/CCR8 in EB formation. Immunosuppression by mTregs was observed by the addition of mTreg supernatant into T cells obtained from mouse splenocytes (data not shown) demonstrating that mTregs have a capacity for both immunosuppressive as well as anti-proliferative capacity.

### CCR8 and FoxP3 expression on EBs/mTregs demonstrate discrete sub-localization

**Fig 6:**
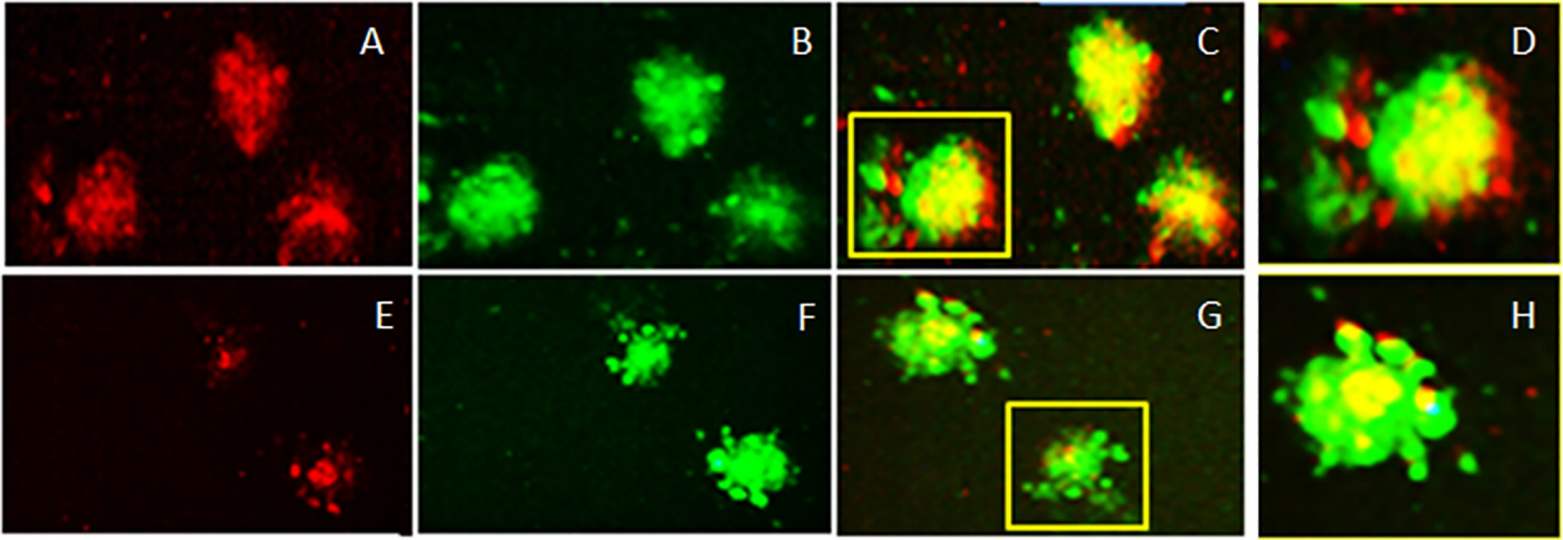
Embryoid bodies/mTreg derived from rat MSCs express CCR8 and FoxP3. CCR8 (6A, red) and FoxP3 (6B, green) co-localize (6C, yellow; 10x mag and 6D, 20X mag). This expression of CCR8 is inhibited by a monoclonal antibody against CCL1 (> CCL1) [6E-G; 10Xmag; H,20X Mag].

Since mTregS /EBs express CCR8 we tested ligand specificity of this receptor and co-localization with FoxP3. It was found that EBs/mTregs derived from rMSCs express CCR8 (Fig 6A, red) and FoxP3 (Fig 6B, green) which co-localize (Fig 6C, yellow; 10xmag and 6D, 20x mag). Inhibition by an anti—CCL1 antibody (>CCL1) blocked CCR8 expression but not Foxp3 expression; showing that CCL1 interacts with its receptor CCR8 on mTregs (Fig 6E and 6F). The expression of CCR8 and FoxP3 is not uniform but localized in sub-regions of the EB [(Fig 6C and Fig 6G; 10x mag) (Fig 6D and Fig 6H; 20x mag)]. Interestingly, CCR8 is expressed in the leading edge (moving towards chemokine CCL1 gradient) of the mTregs EB suggesting its role in directing the cells to the site of injury. CCR8 also co-localizes with Foxp3 in the center of the EB, whereas FoxP3 predominantly occurs in the rear suggesting differential expression of these receptors on EBs/mTregs suggesting discrete localization which may enable specific antigen response. CCR8 expression at the leading edge is significantly abrogated in the presence >CCL1 suggesting an essential role of CCL1/CCR8 in homing of mTregs to the site of injury.

## Discussion

We have developed a method to induce and expand EBs from stress induced MSCs, isolated a novel subtype of regulatory T cells with immunosuppressive properties and have identified the chemokine CCL1 and its receptor CCR8 as modulators of this function. When rat MSCs were exposed to stress (serum removal) or inflammatory microenvironment, they formed colonies or EBs which express Treg specific markers CD25 (IL2 receptor alpha chain) and FoxP3 (transcription factor forkhead box P3). Specifically, MSC derived Tregs or mTregs are obtained from serum deprived rat MSCs (rMSCs), injured smooth muscle cells (SMC) + rMSC and injured endothelial cells (EC) + rMSCs co-cultures. Adherent non-colony forming MSCs do not express these markers. These MSC derived EBs show self-renewal capacity and are multipotent as observed by their ability to differentiate into chondrocytes, adipocytes and neurons which has relevance in designing both MSCs and Treg based therapies in human diseases.

Our findings that non adherent MSCs have a capacity to form spheres or EBs as those reported for embryonic stem cells opens up novel avenues for research and therapy. While ESCs and IPSCs are known to make EBs, this has not been previously described for MSCs. In addition, the identification of CC chemokine CCL1 receptor CCR8 on EB subsets offers an explanation for the mechanism of recruitment and homing of these cells to the site of injury. CC chemokine CCL1 is the known ligand for CCR8. CCR8 is involved in the regulation of the Th1-Th2 response and is known to be expressed in different subsets of Tregs. We have found CCR8 to be a functional receptor on rMSCs as its specific ligand CCL1 increases proliferation of non-adherent cells after migration across a chemotactic chamber *(Haque et*. *al*. *2016 unpublished observations)*. Thus CCL1/CCR8 may play an important role in migration; homing and proliferation of MSC derived cells and mTregs/EBs.

Treg defects have been discovered in patients with multiple sclerosis (MS), type I diabetes (T1D), psoriasis, myasthenia gravis (MG) and other autoimmune diseases [13]. Similar links may also exist for atopy, allergic diseases [14] and cardiovascular disorders such as atherosclerosis [15]. Several studies support Foxp3-positive regulatory T cells (Tregs) as inhibitors of atherosclerosis; however, the mechanism underlying this protection remains elusive. A reduced *in vitro* immune suppression of the patient's Treg cells seems to be the basis for these diseases. This has led to an increasing interest in the possibility of using Tregs in immunotherapy to treat or prevent chronic infections, autoimmune diseases, allergies and transplantation-related complications, such as graft rejection or graft-versus-host disease (GvHD) [16]. Treg therapy has been shown to be well tolerated in patients receiving stem cell treatment and its immune-modulatory effect in clinical setting is under investigation [17–18]. While MSC and Tregs may utilize similar mechanisms of immunomodulation, each also has distinct properties which need to be examined. MSc derived mTregs may open up be a link to the distinct function of Treg mediated immunesuppression in diverse environments.

In this study we show that mTregs exhibit distinct immunomodulatory and remodeling properties when co-cultured with injured SMCs or Huvecs. This was observed SMCs) / Huvecs were subjected to scratch assay and co- cultured with either MSCs or mTregs. Both SMCs and Huvecs quickly recovered after injury in the presence of MSCs; as observed by the relative wound confluency (RWC). However, this was accompanied by the invasion or relative wound density (RWD) of cells leading to increased cell proliferation in the presence of MSCs and aberrant wound repair. When SMC/Huvecs were co-cultured with mTregs, the RWC remained intact; however, the RWD was inhibited showing that mTregs modulate immunosuppression. The mTreg specific EBs when re–introduced into the inflammatory microenvironment have inhibitory capacity. For example, when non-adherent FOXP3+ mTregs are added to injured SMC or endothelial cells, significant decrease in cell migration, invasion (relative wound density or RWD) and proliferation (relative wound confluency or RWC) is observed. On the other hand, when MSC is added alone, there is complete wound closure and higher RWC and RWD, suggesting that the conversion of MSC to mTreg is an important factor in wound healing and remodeling.

A stem-cell niche is a spatial structure in which stem cells are housed and maintained by allowing self-renewal in the absence of differentiation. The crosstalk between MSCs and neighboring cells (SMCs/ECs) activate cytokines/ chemokines in the microenvironment facilitating the formation of EBs and mTregs which mediate immunomodulation and vascular remodeling. mTregs influence the phenotypic conversion of interacting cells in response to inflammatory microenvironment. Therefore, the interactions between MSCs with their nearby microenvironment, i.e. the stem cell niche, in healthy *versus* damaged or diseased tissues must be understood in order to design future clinical applications. The restoration of a functional niche will be essential to safeguard durable repair and ensure continual replacement of mature cells lost to physiological turnover or subsequent stress or damage. Our novel findings expand upon current therapies that rely upon MSCs and/or Tregs by employing MSC specific EBs to produce niche specific mTregs for various downstream applications.

There are certain limitations to this study as target antigens and mTreg subtypes remain to be elucidated. Identification of antigen specific mTreg subtypes will lead to development of methods for designing specific therapeutic regimens and targeting specific cell and tissues. Drug interaction, combination therapy with mTregs needs to be elucidated which may identify novel pathways and insights into stem cell biology result in beneficial outcomes. Therefore, MSC derived EBs/mTregs needs further investigation. Studies in our lab are underway to characterize MSC derived EBs and in the identification of mTreg subtypes/target antigens.

In conclusion, it is proposed that MSCs have the capacity to form EBs and generate mTregs that have immune—modulatory and regenerative capabilities. As mTregs subtypes are generated in response to specific microenvironment, they are antigen/tissue specific and therefore capable of more precise targeting as opposed to direct MSC or peripherally obtained Treg treatments. This targeted approach has the potential for development of adoptive transfer methodologies which has more chances for success. Our method for the conversion of MSCs to EBs and mTregs in an inflammatory microenvironment offers a unique process for targeted stem cell therapy. To the best of our knowledge, MSC derived Tregs (mTregs) and their functionality have not been previously described. MSC derived immune-modulators will provide an alternate option to current therapies and is expected to have fewer side effects and thus a higher potential for therapeutic use. In addition, MSC derived EBs may serve as a replacement for embryonic stem cell derived EBs for tissue engineering with fewer ethical concerns. Thus, this method has far reaching applications in research, diagnosis and treatment.

## Supporting information

S1 Data Real time video of cell migration in injured human endothelial cells (ECs) subjected to MSCs or mTregs; each experiment is shown in triplicate (MSCs:T1, T2,T3 or mTregs:T1,T2,T3). (ZIP)

S2 Data Real time video of cell migration in injured human smooth muscle cells (SMCs) subjected to MSCs or mTregs; each experiment is shown in triplicate (MSCs:T1,T2,T3 or mTregs:T1,T2,T3). (ZIP)
